# Phase Structure Recording in a Nematic Side-Chain Liquid-Crystalline Polymer

**DOI:** 10.3390/polym12020356

**Published:** 2020-02-06

**Authors:** Ivan Budagovsky, Aleksey Kuznetsov, Sergey Shvetsov, Mikhail Smayev, Alexander Zolot’ko, Alexey Bobrovsky, Natalia Boiko, Valery Shibaev

**Affiliations:** 1 P.N. Lebedev Physical Institute, Leninsky pr., 53, Moscow 119991, Russia; aleksey_kuzn@mail.ru (A.K.); shvetsov@polly.phys.msu.ru (S.S.); smayev@lebedev.ru (M.S.); zolotko@lebedev.ru (A.Z.); 2 Faculty of Physics, Lomonosov Moscow State University, Leninskie gory, Moscow 119991, Russia; 3 International Centre of Laser Technology, Mendeleev University of Chemical Technology of Russia, Miusskaya pl., 9, Moscow 125047, Russia; 4 Faculty of Chemistry, Lomonosov Moscow State University, Leninskie gory, Moscow 119991, Russia; bbrvsky@yahoo.com (A.B.); boiko2@mail.ru (N.B.); lcp@genebee.msu.ru (V.S.)

**Keywords:** liquid-crystalline polymer, optical nonlinearity, supramolecular structure, optical recording, Fréedericksz transition

## Abstract

Dye-doped nematic side-chain liquid-crystalline polymers possess extraordinary large optical nonlinearity and ability to store the induced orientational deformations in a glassy state, which makes them a very promising material for photonic applications. In this study, the phase structures were generated and recorded in the bulk of a 50-μm layer of a nematic liquid-crystalline side-chain polymer, containing polyacrylate backbone, spacer having five methylene groups, and phenyl benzoate mesogenic fragment. The polymer was doped with KD-1 azodye. The director field deformations induced by the light beam close to the TEM_01_ mode were studied for different geometries of light–polymer interaction. The phase modulation depth of 2π was obtained for the 18-μm spacing between intensity peaks. The experimental data were analyzed based on the elastic continuum theory of nematics. The possibility to induce and record positive and negative microlenses in the polymer bulk was shown experimentally.

## 1. Introduction

External fields can greatly affect the supramolecular structure and molecular ordering of soft matter. The most known example is the transformation of the director field in the nematic liquid crystals (NLCs) under action of the electromagnetic field. This effect is widely used in display applications, where the NLC director is rotated by an AC electric field. This rotation modulates the phase shift for the extraordinary wave and, as a consequence, the intensity of light passed through crossed polarizers [1].

The supramolecular structure of NLCs can also be easily transformed by the light field. This effect can be due to either the optical anisotropy [2,3] or modification of intermolecular forces upon excitation of dyes introduced into nematic host [4,5,6]. Due to the cooperative molecular reorientation, the orientational optical nonlinearities of NLCs are extremely high. The nonlinearity of optically transparent NLCs is nine orders of magnitude higher than the Kerr nonlinearity of CS_2_ [7]; it can be increased by two orders of magnitude by dye doping [4]. Among the wide variety of nonlinear optical phenomena in NLCs, there are aberrational self-focusing [8,9] and self-defocusing [10], formation of light waveguides [11,12], light induced gratings [13], and pattern formation [14]. All these phenomena are related to various structures of deformed director field.

When an external electromagnetic field affecting NLCs is turned off, the NLC director field relaxes to the uniform state (an exception is the photoalignment, i.e., the optical modification of the orienting properties of the surfaces of NLC layers [15]). The deformed director field can be stored in the NLC bulk by a photoinduced polymer network in the nematic matrix. In such a way, the periodic (photonic) structure [16] and the structure with large in-plane refractive-index gradient [17] were recorded.

Another approach is the creation of spatially nonuniform polymer network in an undeformed NLC. In this case the steady-state inhomogeneous NLC structure reveals itself upon application of external ac field. This method was used to record conventional [18,19,20,21,22] and Fresnel [23,24,25] lenses. Phase structures, including microlens arrays [26], can be recorded in amorphous polymers. In this case, sufficiently high absorption coefficient is usually required.

It is known that side-chain liquid-crystalline polymers exhibit various phases, including the nematic one [27]. As was recently found, the nonlinear optical properties of nematic liquid-crystalline polymers (NLCPs) are similar to those of low molecular weight nematics. Transparent samples have positive nonlinearity coefficient at the level n_2_~10^−4^ cm^2^/W [28]. However, due to the high viscosity of the polymer matrix, which slows down the rotational diffusion, the dye-doped NLCPs in the nematic phase have very high optical nonlinearity—of the order of 10^−1^ cm^2^/W at a dye concentration of 0.05% [29], which is two orders of magnitude higher than that for the low molecular weight matrix at the same dye concentration. These properties make it possible to induce the director deformations in the nematic state and preserve them in the glassy state, using relatively low light intensities. In contrast to the polymerization technique, this process is reversible, since the induced director deformations are erased upon transition from glassy to nematic state.

The nonlinear optical response of NLCPs, similar to that of low-molar-mass NLCs, is highly nonlocal, i.e., the angle of NLCP director rotation at a given point is determined by not only the light intensity at this point, but also the intensity distribution in a vicinity of this point. For this reason, the recorded light induced deformation field can greatly differ from the light intensity distribution. In addition, the light induced NLCP director field distortions can relax and even transform during cooling to glassy phase.

In our previous study [30], we recorded the images of the bell-like (Gaussian) intensity profiles at the normal light incidence on the NLCP layer, in which case the threshold light-induced Fréedericksz transition occurred. It was found that the size of the deformed region is no less than the NLCP layer thickness independent of the light beam waist width.

In this paper, in order to elucidate the possibilities of generation and recording of complex phase profiles, we study the NLCP director field deformation under action of light beams of various sizes with oscillating intensity profiles using different geometries of light–NLCP interaction and further conservation of deformed director state.

## 2. Materials and Methods

### 2.1. Nematic Liquid-Crystalline Polymer Cell

As an experimental material we used the side-chain polymer, containing polyacrylate backbone, spacer having five methylene groups, and phenyl benzoate mesogenic fragment (PAA). Its structural formula is 
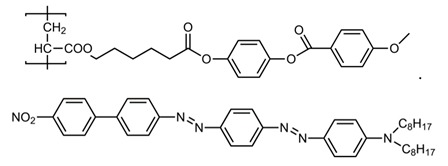


The PAA polymer (the polymerization degree is 22) was doped with azo dye KD-1 with a concentration of 0.05 wt %. This NLCP has a nematic phase at temperatures below 123 °C and transforms into the glassy state at *T* < 26 °C. In the nematic phase, the NLCP sample exhibits a high negative optical nonlinearity (*n*_2_ ~ 0.1 cm^2^/W) [29]. The NLCP material was filled into the glass cell and cooled to room temperature within a few hours. The cell thickness was set by 50-μm Teflon spacers. To provide uniform planar alignment of the NLCP film, the inner surfaces of glass plates composing the cell were coated by polyimide and rubbed in opposite directions. The pretilt angle value was measured to be less than 1°. The optical anisotropy (the difference in the refractive indices of the extraordinary and ordinary waves) was found previously to be δ*n*_max_ = 0.08 for a temperature of *T* = 105 °C and δ*n*_max_ = 0.12 for a temperature of *T* = 22 °C [30]. The absorption coefficients, measured with an MS-122 spectrophotometer (Proscan Special Instruments, Minsk, Belarus), were 200 and 35 cm^−1^ at the wavelength λ = 532 nm for the extraordinary and ordinary waves, respectively.

### 2.2. Experimental Setup

**Figure 1:**
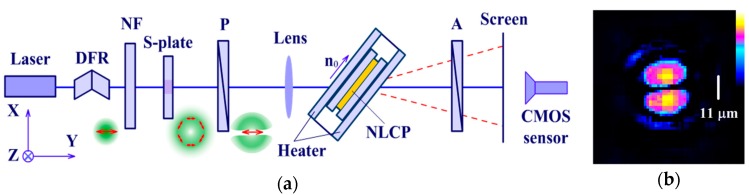
(**a**) Schematic of the experimental setup (top view): (DFR) double Fresnel rhomb, (NF) neutral density filter, (P) polarizer, (A) analyzer–polarizer. The vector **n**_0_ is the unperturbed director. S-waveplate is oriented to produce azimuthal polarization. Red double arrows indicate the polarization direction. (**b**) Color map of the beam intensity distribution in the focal plane. White bar indicates the distance *d* between intensity peaks.

Phase structures were recorded by a beam with the intensity distribution close to that of TEM01 mode. To obtain such an intensity distribution, the linearly polarized Gaussian light beam from a 532L-11B DPSS laser (λ = 532 nm, Integrated Optics, Vilnius, Lithuania) was passed through an RPC-515-4 S-waveplate (Laserand, Montreal, QC, Canada) and a polarizer (Figure 1a). Then it was focused by a lens with a focal length of 38 mm onto the sample. The double Fresnel rhomb and a set of neutral filters made it possible to change the polarization of the radiation incident on the S-waveplate and adjust the radiation power. The sample could be rotated about the vertical axis (the director of the NLCP was in the horizontal plane, i.e., located in the plane of light beam incidence). The temperature of the sample was maintained by a heater, made in the form of two additional glass plates with indium tin oxide (ITO) coating on the outer surfaces, connected to an HY-3003-2 DC power supply (Mastech, Weinan, China). The spatial distribution of the beam intensity in the focal plane (Figure 1b) and the aberrational pattern in the far field were recorded using a CMOS camera based on the IMX179 matrix (Sony, Tokyo, Japan). In the focal plane, the beam size at a level of 1/e2 of the intensity maximum (for a vertical cross-section profile drawn along the two maxima of the beam) was about 30 μm, the distance between maxima was d = 11 μm, and effective mode area was Aeff = 1500 μm2.

### 2.3. Methodics

An increase in the NLCP temperature leads to a decrease in the polymer viscosity, and with it a decrease in the magnitude of orientational optical nonlinearity [30]. To record the phase structure in the sample, the NLCP layer was heated to *T* = 105 °C (the accuracy of temperature measurements was 1 °C). This temperature ensured the optimal balance between the rate of director reorientation (phase structure formation) and the nonlinearity value. The heated sample was irradiated for a time sufficient to form a steady-state director field deformation. This time depends on the geometry of the experiment. At normal light incidence (α = 0°, threshold geometry), the required exposure time was hours, while at an oblique incidence (non-threshold geometry, α = 35°) it was tens of minutes. The light-induced director deformation results in a formation of the aberrational ring pattern in the far field (effect of aberrational light self-action) [10]. We used this pattern to control the recording process. After the development of the steady-state aberrational pattern, the heater was turned off, and 30 s later the radiation was turned off as well. Such procedure made it possible to minimize changes in the induced refractive index, compensating for two oppositely directed effects: an increase in sensitivity to light at lower temperature and the director relaxation in the absence of illumination during the transition to the glassy state.

The recorded phase structure was observed in crossed polarizers at an angle of 45° to the NLCP director with an L-213 POLAM microscope (LOMO, Saint Petersburg, Russia) using a 525-nm narrow-band interference filter. The phase profiles were determined in the cross section passing through both peaks of the light beam (in the plane perpendicular to the unperturbed director) by positions of the bright and dark regions in the microscopic texture [30]. Note that the cooling of the sample also leads to an increase in the optical anisotropy for the entire matrix (about 50%); therefore, the phase change δ*S* is proportionally larger than that occurring during the formation of the aberrational pattern and estimated from the number of aberrational rings.

Reheating the NLCP to the nematic phase led to complete relaxation of the recorded director field and erasing the phase structure.

## 3. Results and Discussion

We shall present the experimental results separately for normal and oblique light incidence on the NLCP sample. In the first case the director reorientation is of the threshold type (the Fréedericksz effect), while in the second case the director should rotate at any light power.

### 3.1. Normal Light Incidence

At normal incidence of the light beam, the director deformation and related light-beam broadening has a pronounced threshold character, since there is practically no pretilt (less than 1°) in the NLCP cell. The value of the Fréedericksz transition threshold power at the beam focus was Pth = 30 μW (the threshold intensity Ith = Pth/Aeff was 2 W/cm2). In what follows, we will use the dimensionless light beam power normalized to the threshold power in the focus, δ = P/Pth.

**Figure 2:**
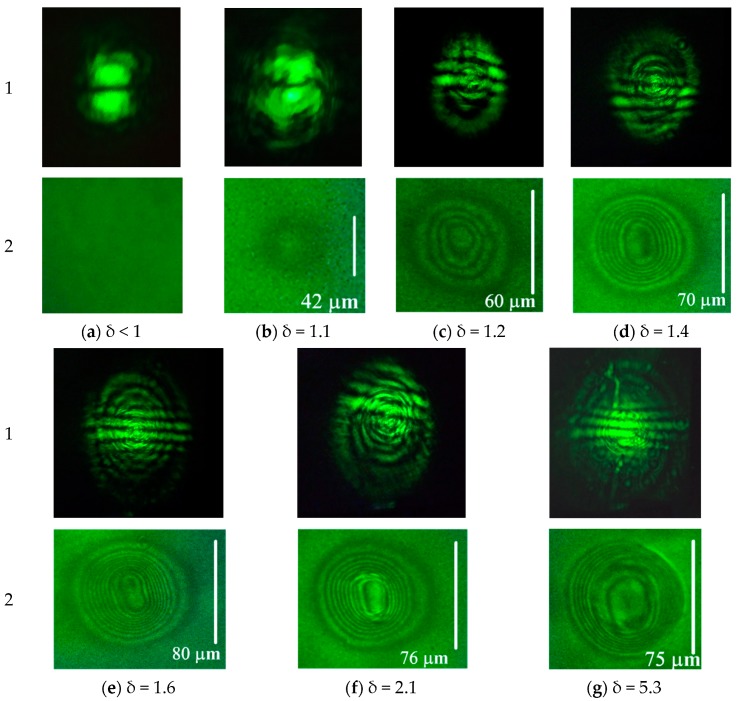
Steady-state aberrational patterns for the normal (α = 0°) light incidence (row “1”) and corresponding microphotographs (row “2”) obtained at the focal plane for normalized beam power: δ = (**a**) 1, (**b**) 1.1, (**c**) 1.2, (**d**) 1.4, (**e**) 1.6, (**f**) 2.1, and (**g**) 5.3. Aberrational-pattern vertical angular size is (**a**) 0.07, (**b**) 0.09, (**c**), (**d**) 0.54, (**e**) 0.64, (**f**) 0.64, and (**g**) 0.9 rad. Vertical bar indicates the size of the recorded phase structure.

At powers below the threshold, no changes occurred in both the light beam intensity in the far-field (Figure 2a1) and the refractive index of the extraordinary wave (Figure 2a2).

A slight excess over the threshold power (δ = 1.1) led to the formation of a region of weak director deformation corresponding to the nonlinear phase shift |δS| = 2π and a change in the refractive index of the extraordinary wave |δn| = 0.01 (Figure 2b2). The light intensity pattern in the beam was somewhat distorted (Figure 2b1). The size of the recorded image was 42 μm; two regions corresponding to beam maxima were not resolved in the phase pattern.

An increase in the light beam power to δ = 1.2 results in the formation of an aberrational pattern, showing a larger change in the refractive index. In this case (Figure 2c1), horizontal interference fringes with a spatial period of 28 mm (at a distance of *D*_s_ = 56 cm from the cell to the screen) appear in the pattern. This size corresponds to the spatial period $\Lambda_{s}=\lambda D_{s}/d$, formed by the interference of two coherent sources located at a distance d from each other in the vertical plane. For the spacing of the intensity maxima d = 11 μm in the beam, the use of the above formula yields $\Lambda_{s}=27$ mm, which practically coincides with the experimental value. For the appearance of the fringes, the director deformation should be higher than a certain value at which the introduced wave front curvature allows two parts of the beam to overlap each other in the far field. These interference fringes allow one to control the distance between the intensity peaks in the incident beam. The phase change |δS| determined from microphotograph (Figure 2c2) was 8π (|δn| = 0.03). In the central region, the pattern is noticeably elongated along the axis passing through the beam maxima, but the regions corresponding to the incident beam peaks were not resolved (Figure 2c2).

At δ = 1.4, the induced phase variation increased to |δS|=16π (|δn| = 0.06), the internal region with weakly varying phase is clearly seen (Figure 2d2). A further increase in the light power to δ = 1.6 enhanced the self-action (Figure 2e1); in the recorded picture of the phase structure, the elongated region with slowly varying phase was more pronounced (Figure 2e2). The phase change amounts to 20π (|δn| = 0.08).

It should be noted, that the maximum phase shift *S*_max_ corresponding to the complete director orientation by 90°, estimated by the formula $\left|\delta S_{\max}\right|=2\pi \left|\delta n_{\max}\right|L/\lambda$, amounts to 24π. Thus, at δ = 1.6 the nonlinear phase shift is close to the saturation.

A twofold excess of the threshold value (δ = 2.1) leads to a distortion in the central part of the recorded phase structure and the beginning of the formation of a plateau with the maximum director reorientation (Figure 2f2). The maximum light-induced phase shift remained near 20π.

Finally, a significant increase in power (δ = 5.3) results in the formation of an aberration pattern with a much brighter central part (Figure 2g1). This is related to the corresponding phase distribution (Figure 2g2) with a large plateau area in the central part, providing lower divergence for the most intense rays of the light beam. Note that such a recorded phase distribution is quite similar to that in the case of the action of a Gaussian beam under the same experimental conditions [30].

We emphasized that for the above cases, except for the last one, the recorded structure is elongated to a certain degree. However, the two peaks in the light beam intensity distribution were not resolved in the recorded phase structure.

These peaks began to be resolved with a slight increase in the distance between them. This can be achieved by the displacement of the cell from the focal plane.

**Figure 3:**
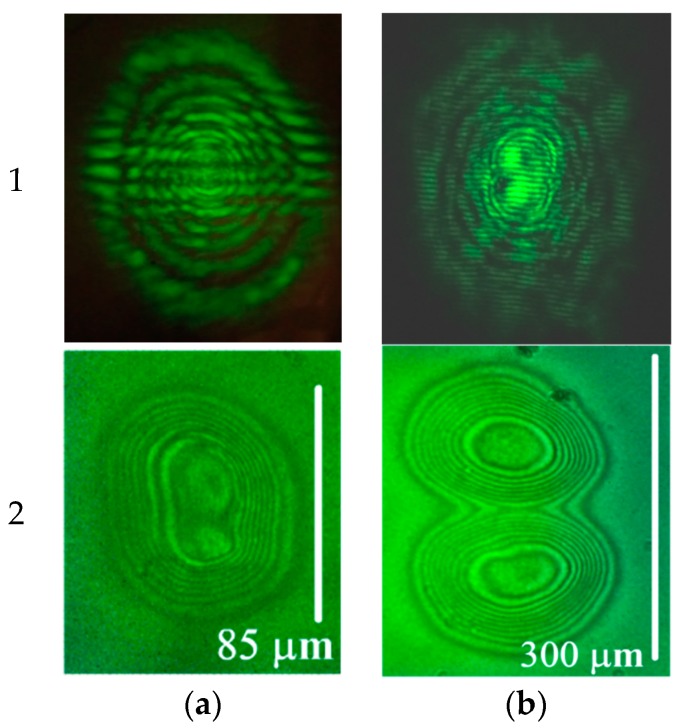
Steady-state aberrational patterns for the normal (α = 0°) light incidence (row “1”) and corresponding microphotographs (row “2”) obtained for normalized beam power δ = (**a**) 5.3 and (**b**) 10.6. The cell position is shifted from the focal plane by (**a**) 1 mm (*d* = 18 μm) and (**b**) 5 mm (*d* = 120 μm). The aberrational-pattern vertical angular size is (**a1**) 0.5 and (**b1**) 0.3 rad. Vertical white bar indicates the size of the recorded phase structure.

At a distance of Δ*y* = 1 mm from the focal plane, the peak spacing becomes 18 μm. With the sample at this position, an aberrational pattern (Figure 3a1) is formed with a smaller spacing of fringes (17 mm). The intensity peaks were confidently resolved in the phase structure (Figure 3a2).

At Δ*y* = 5 mm, with a larger beam size (d = 120 μm), not only the distance between the peaks in the beam intensity coincides with the distance between the phase shift maxima, but also the shape of the beam resembles the recorded phase structure (Figure 3b2).

**Figure 4:**
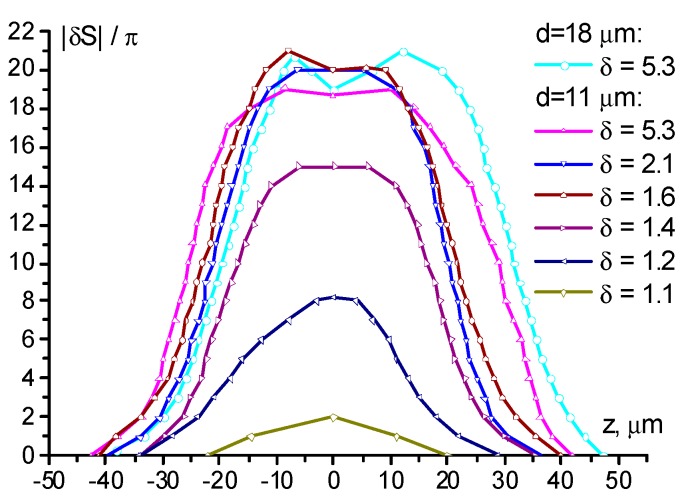
The phase shift reconstructed from the microphotographs shown in Figure 2b–g and Figure 3a. Lines are an eye-guide connecting the experimental points.

The reconstructed phase (Figure 4) allows the estimation of the maximum gradient ∂S/∂z of the phase shift *S* along the *Z*-axis (see Figure 1) at different director field deformations. This value determines the divergence of the aberrational pattern $\theta \left(z\right)=\frac{\lambda }{2\pi }{\left(\left|\frac{\partial S}{\partial z}\right|\right)}_{\max}$ (where *S* should be corrected for the temperature variation). With an increase in the phase shift amplitude from 2π (for δ = 1.1) to 20π (for δ = 2.1), the maximum value of |∂S/∂z| increased from 0.1 to 2 π/μm. The value |∂S/∂z|=2 π/μm was retained for all cases when deformation was saturated and reflected the limiting rate of phase shift change, which was allowed by elastic forces at a given thickness of the NLCP layer.

For the recording of complex structures, the resolution of neighboring deformation peaks is most important. As a criterion for the resolution of the phase structure, we can take a change in the phase shift between peaks in excess of 2π. This situation is realized for intensity peak spacing d = 18 μm (Figure 3a2). As follows from the beam intensity distribution, the actual width of intensity dip, governing the phase variation between the peaks, is much lower: for 18 μm spacing it is about 8 μm at the 90% intensity level. Thus, the value of 8 μm can be treated as a characteristic parameter of the spatial resolution of the recording for the given cell.

### 3.2. Oblique Incidence

**Figure 5:**
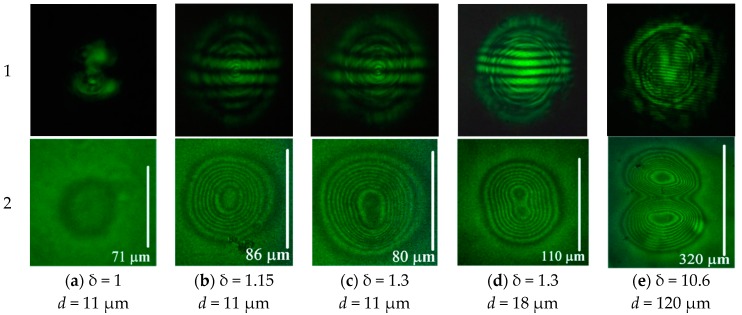
Steady-state aberrational patterns for the oblique (α = 35°) light incidence (row “1”) and corresponding microphotographs (row “2”) obtained for normalized beam power: at (**a**–**c**) focal plane (d = 11 μm), (**d**) 1-mm displacement from the focal plane (d = 18 μm), and (**e**) 5-mm displacement from the focal plane (*d* = 120). The normalized power is δ = (**a**) 1, (**b**) 1.15, (**c**,**d**) 1.3, and (**e**) 10.6. Aberrational-pattern vertical angular size is (**a**) 0.1, (**b**) 0.34, (**c**) 0.37, (**d**) 0.36, and (**e**) 0.22 rad. Vertical white bar indicates the size of the recorded phase structure.

**Figure 6:**
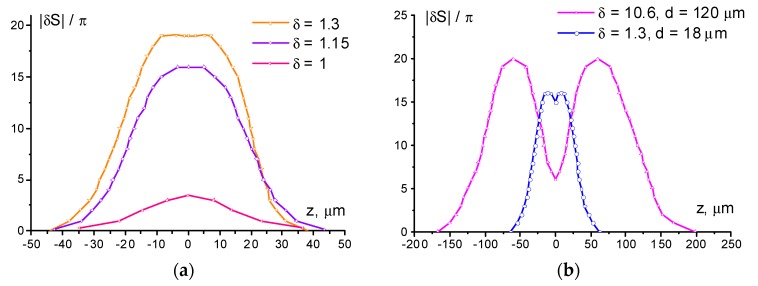
The phase shift reconstructed from the microphotographs shown in Figure 5 for *d* = (**a**) 11 and (**b**) 18 and 120 μm. Lines are an eye-guide connecting the experimental points.

At oblique incidence, the light-induced deformation occurred much faster (10–30 min). As in the case of normal incidence, when recording the light-induced deformation at the focus of the lens (*d* = 11 μm), the beam peaks were not resolved in the phase structure, regardless of the beam power (Figure 5a–c and Figure 6a). However, with increasing power, the structure of the recorded beam revealed itself. Thus, at a low power of the beam (δ = 1), there was no asymmetry in the phase picture (Figure 5a2); with an increase in the light power, the elongation was noticeable in the central region (Figure 5b2); and with a further increase in δ, two regions began to appear in the central part of the phase structure (Figure 5c2).

Similar to the case of normal light incidence, the peaks of the recorded beam started to resolve with an increase in peak spacing. At d = 18 μm, the positions of the peaks show up (Figure 6b); however, the maximum depth of phase modulation (at δ = 1.3) between them was slightly lower than the chosen resolution criterion of 2π (Figure 5d2).

**Figure 7:**
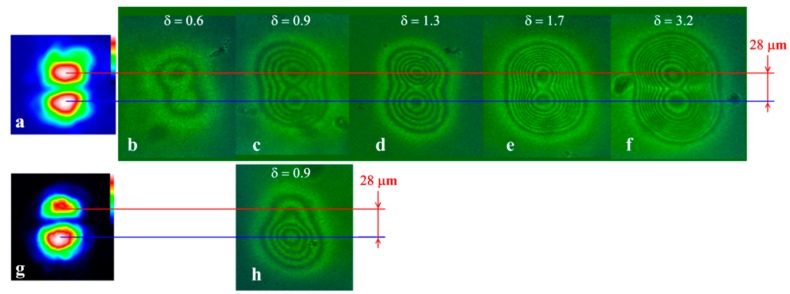
Microphotographs in the case of (**a**) symmetrical and (**g**) asymmetrical beam intensity for normalized beam power: δ = (**b**) 0.6, (**c**) 0.9, (**d**,**h**) 1.3, (**e**) 1.7, and (**f**) 3.2. Oblique incidence (a = 35°). The distance between the intensity peaks is d = 28 μm.

**Figure 8:**
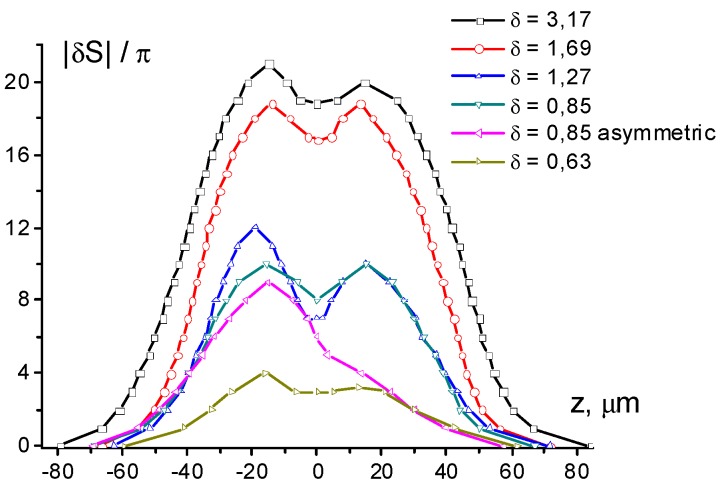
The phase shift reconstructed from the microphotographs shown in Figure 7. Lines are an eye-guide connecting the experimental points.

The phase patterns for the beam of a larger size (d ≈ 28 μm) show a similar character of the dependence on the power (Figure 7). The resolution criterion was satisfied at δ ≥ 0.9 (Figure 7c–f). The situation changes if the intensities of the two peaks of the beam differed significantly. Such a beam was obtained by vertically shifting the S-waveplate with respect to the incident beam (Figure 7g). When recording the beam with the peak intensity ratio 1:1.5, the peak with lower intensity ceased to be resolved in the phase pattern (Figure 7h), which is also clearly seen when comparing the phase profiles (Figure 8).

As for the normal light incidence, with a significant increase in the size of the beam, the phase image shows not only the positions of the peaks but also the shape of the beam (Figure 5e2). This mode of the light effect can be considered in the local approximation without taking into account transverse elastic forces.

Summarizing the above results we could conclude that the phase structures recorded under action of normally and obliquely incident light beams with over-threshold power are quite similar. At the same time, the oblique incidence allows one to record developed phase structures (|δS|~10) at light power below the Fréedericksz transition threshold.

The longer recording times for the normal light beam incidence are related to the fact that at this light–NLCP interaction geometry the optical torque for the unperturbed director field is zero. The director field transformation starts from the growth of a suitable fluctuation, whose occurrence can take some time.

### 3.3. Microlens Recording

**Figure 9:**
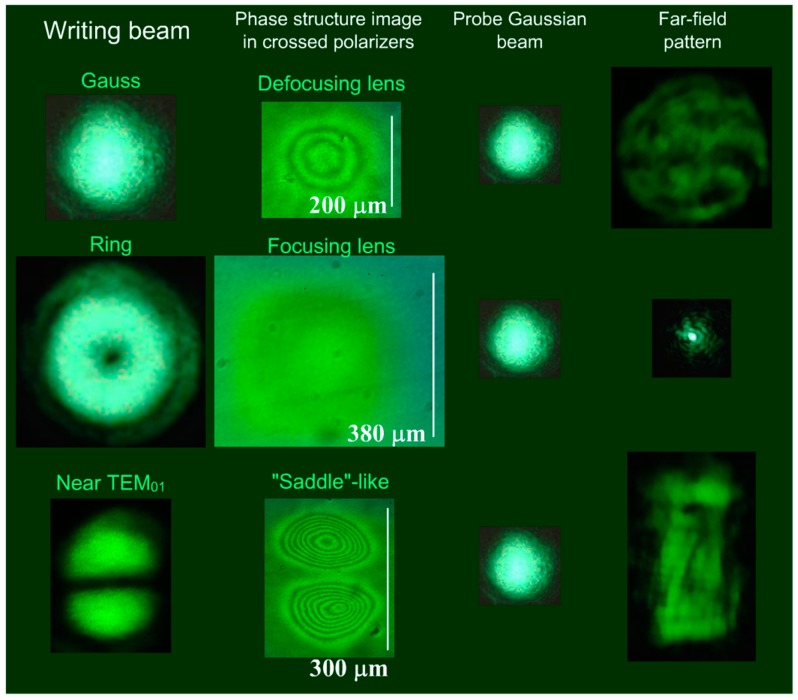
Demonstration of the possibilities of optical recording with different shapes of the light beam (δ = 4.3). First row: Gaussian beam (2*w* = 200 μm) induces a nonlinear phase structure, which acts as a defocusing lens; the divergence of the probe Gaussian beam, passing through the central part of the structure, increases. Second row: ring-shaped beam (beam size ≈ 350 μm) induces a ring-shaped phase structure, which acts as a focusing lens for the probe beam, passing through the central part of the structure. Third row: a TEM_01_-like beam (vertical beam size ≈ 300 μm) induces a phase structure with a saddle-like profile in its central part; probe beam, passing through this region undergo a complex deformation, acquiring a rectangular form. The probe beam and aberration patterns are given on a single scale (the divergence of the Gaussian beam ≈ 0.07 rad).

Various methods of producing lenses were reported for low molecular weight NLCs (including creation of polymer networks) [18,19,20,21,22,23,24,25,31,32] and amorphous polymers [26]. In this study, we applied the optical recording of the phase structures in the NLCP for the formation of negative and positive lenses. In the studied case of the negative nonlinearity, the director rotated normally to the light field **E**, decreasing the refractive index proportionally to the intensity of the light. Recording the phase structure with a relatively wide Gaussian beam (beam diameter 2*w* = 200 μm, δ = 4.3) allowed us to form a phase profile, which acts as a defocusing lens for the beam, passing through its central part (Figure 9, first row). The estimation of the focal length of the induced lens from the microscopic pattern yielded the value of 7 mm.

For negative nonlinearity, it is impossible to record the focusing lens using a light beam with the bell-shaped intensity profile, since the influence of light could only reduce the refractive index. However, it was possible to record the positive lens with a ring-shaped beam: in this case, the refractive index at the beam axis would be higher than at the periphery. Experimentally, the ring-shaped intensity distribution was achieved with an optical vortex, generated by an S-waveplate illuminated with a circularly polarized beam (in this case, the λ/4-plate was placed in front of the S-waveplate). The ring-shaped phase structure, recorded with such a beam (beam size ≈ 350 μm, δ = 4.3) acts as a focusing lens, decreasing the transverse size of the divergent probe beam, passing through its central region (Figure 9, second row).

More complex beam transformations can be obtained by the illumination of different sections of the phase profiles recorded with a beam close to the TEM_01_ mode. For example, the phase structure, recorded with the TEM_01_ beam (vertical beam size ≈ 300 μm, δ = 4.3), will have a saddle-like profile between the peaks. The probe Gaussian beam passing through this region acquires a shape close to rectangle (Figure 9, bottom row).

The recorded light-induced refractive index modification affected only the extraordinary wave. There were no changes in divergence for the ordinary-wave polarization of the probe beam, passed through the recorded structure, i.e., the recorded phase structures were polarization-sensitive.

### 3.4. Estimation of the Director Field Deformation

To estimate quantitatively the light-induced NLCP director field deformation under a light beam with two peaks, separated by the distance Λ, we used the simplest model of one-dimensional periodic light intensity distribution (see Appendix A).

For the contrast κ of the phase pattern, i.e., the ratio of the difference ΔS between maximum and minimum phase shifts and their averaged value $\bar{S}$, we derived a very approximate relation

(1) $$\kappa =\frac{\Delta S}{\bar{S}}\approx \frac{4}{a^{2}},$$

where the parameter a=2L/Λ.

The character of the dependence (1) agrees with the experimental data. At Λ=11 μm (a=9.1), there were no peaks in the image that would correspond to the two light intensity peaks at both the normal and oblique incidences. That is, the light intensity profile has no valley, i.e., the light peaks are not resolved. It should be noted that the plateau area observed in the central part of Figure 2g2 corresponded to vanishing spatial phase variation ΔS (A24) and contrast κ (A26). This situation corresponded to the saturation of the NLCP reorientation according to (A20).

At larger Λ=18 μm (a=5.6), at the normal incidence the contrast is κ≈0.1 at δ=5.6. At the oblique incidence, κ≈0.2 and 0.1 at δ=1.4 and 3.2, respectively. The estimate (1) κ≈0.13 agrees with these experimental values in the order of magnitude. At Λ=32 μm (a=3.1), we observed higher contrast κ≈0.5 at δ=1.3. For large Λ=120 and 130 μm (a=0.83 and 0.76), the intensity peaks were well resolved. However, the pronounced optical response nonlocality was still manifested.

## 4. Conclusions

We studied the reversible optical phase structure recording in the dye-doped nematic side-chain liquid-crystalline polymer under action of the light beam with an oscillating intensity profile, presented by the TEM_01_-like mode. For the studied thick NLCP sample (L = 50 μm), the recorded phase modulation between the light intensity peaks as high as 2π was obtained for the beam spacing L/3.

The geometry of the light–polymer interaction did not affect significantly the phase structure recording. However, at the oblique incidence the director deformation was much faster in comparison with the case of normal incidence. The estimations performed within the elastic continuum theory of the NLCs agree with experimental data.

The recording of the phase structures acting as focusing and defocusing polarization sensitive microlenses was implemented using light beams of various shapes.

The obtained results are important for the analysis of the possibilities of the recording of various phase structures (polarization-sensitive gratings, optical waveguides, and other optical elements).

## Figures and Tables

**Figure A1:**
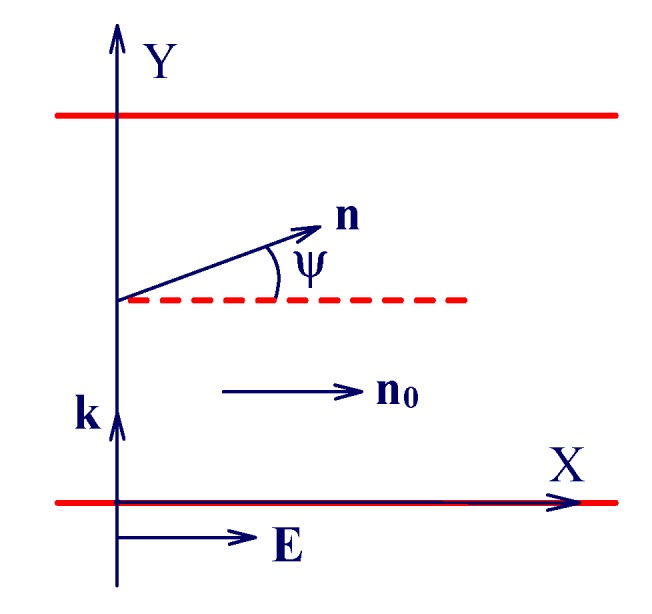
The schematic of the interaction of NLCPwith linearly polarized light beam incident normally on the sample with initial uniform planar alignment characterized by the director **n_0_**. **E** and **k** are the electric field and wave vector of the incident light, and ψ is the angle of the director rotation.

## Appendix A

Let us estimate the effect of light on the NLCP director field for a normal incidence of a light beam. The schematic of light–NLCP interaction is shown in Figure A1. The electric field vector **E,** the wave vector **k**, the unperturbed director **n_0_**, and rotated director **n** are in the horizontal plane XY.

The NLCP director was rotated by the torque $\Gamma_{s}$ being the sum of the torque due to dye molecule excitation and the torque $\Gamma_{\mathrm{tr}}$ due to dielectric anisotropy $\Delta \varepsilon_{\mathrm{opt}}$ of transparent NLCP at the light frequency, which can be represented in the form

(A1) $$\Gamma_{s}=\eta_{s}\Gamma_{\mathrm{tr}},$$

where

(A2) $$\Gamma_{\mathrm{tr}}=\frac{\Delta \varepsilon_{\mathrm{opt}}}{4\pi }\left(nE\right)\left[n\times E\right],$$

and $\eta_{s}$ is the proportionality coefficient (enhancement factor). This factor is negative, which implies the light-induced director rotation away from the light field direction. The light field can be written as

(A3) $$E=\frac{1}{2}\left(e_{p}A\left(y\right)e^{i\left(ky-\omega t\right)}+c.c.\right),$$

where $e_{p}=i$ is the unit polarization vector, A is the light field amplitude.

In order to simulate the light-induced NLCP director field deformation under a light beam with two peaks, i.e., an oscillating intensity profile, we used the simplest model of one-dimensional periodic light intensity distribution

(A4) $$\left|A{|}^{2}=\right|E_{0}{|}^{2}\left(1-\cos\frac{2\pi x}{\Lambda }\right),$$

where $|E_{0}{|}^{2}$ is the average square of light field amplitude and Λ is the spatial period of the light field intensity.

Starting from the torque balance equation and using the calculation procedure implemented in [29], we arrived at the equation describing the NLCP director field transformation in the one elastic constant approximation

(A5) $$\frac{\partial \psi }{\partial \tau }=\psi_{\xi \xi }+\psi_{\eta \eta }+\delta \left(1-\cos a\xi \right)\sin\psi \cos\psi ,$$

where ξ=πx/L, η=πy/L, $\tau =t/\tau_{0}$, $\tau_{0}=\gamma_{1}L^{2}/\pi^{2}K$, $\delta =\left|\eta_{s}\right|\Delta \varepsilon_{\mathrm{opt}}|E_{0}{|}^{2}L^{2}/8\pi^{3}K$, $\gamma_{1}$ is the viscosity coefficient, *K* is the Frank elastic constant, and $a=2\frac{L}{\Lambda }$. The boundary conditions for Equation (A5) are

(A6) ψ(ξ,η=0,τ)=ψ(ξ,η=π,τ)=0.

In compliance with (A6), we assumed the longitudinal dependence of the rotation angle ψ in the form

(A7) $$\psi \left(\xi ,\eta ,\tau \right)=\tilde{\psi }\left(\xi ,\tau \right)\sin\eta .$$

Expanding the trigonometric functions in (A5) in powers of ψ up to the third-order term, substituting (A7) into (A5), multiplying by sinη, and averaging over the longitudinal coordinate η, we find

(A8) $$\frac{\partial \tilde{\psi }}{\partial \tau }={\tilde{\psi }}_{\xi \xi }-\tilde{\psi }+\delta \left(1-\cos a\xi \right)\left(\tilde{\psi }-\frac{1}{2}{\tilde{\psi }}^{3}\right).$$

For the function $\tilde{\psi }$, we assumed the mode approximation

(A9) $$\tilde{\psi }\left(\xi \right)=\psi_{0}\left(\tau \right)-\psi_{1}\left(\tau \right)\cos a\xi ,$$

where $\psi_{0}$ and $\psi_{1}$ are the mode amplitudes. The equations for the amplitudes $\psi_{0}$ and $\psi_{1}$, derived from (A8), have the form

(A10) $$\frac{\partial \psi_{0}}{\partial \tau }=\left(\delta -1\right)\psi_{0}-\frac{1}{2}\delta \psi_{0}^{3}+\frac{1}{2}\delta \psi_{1}-\frac{1}{4}\delta \left[3\psi_{0}^{2}\psi_{1}+3\psi_{0}\psi_{1}^{2}+\frac{3}{4}\psi_{1}^{3}\right],$$

(A11) $$\frac{\partial \psi_{1}}{\partial \tau }=\left[\delta -\left(1+a^{2}\right)]\psi_{1}+\delta \psi_{0}-\frac{1}{2}\delta [\psi_{0}^{3}+3\psi_{0}^{2}\psi_{1}+\frac{9}{4}\psi_{0}\psi_{1}^{2}+\frac{3}{4}\psi_{1}^{3}\right].$$

The linearized system of Equations (A10) and (A11) is

(A12) $$\frac{\partial \psi_{0}}{\partial \tau }=\left(\delta -1\right)\psi_{0}+\frac{1}{2}\delta \psi_{1},$$

(A13) $$\frac{\partial \psi_{1}}{\partial \tau }=\delta \psi_{0}+\left[\delta -\left(1+a^{2}\right)\right]\psi_{1}.$$

The solutions to this system of equations are proportional to $e^{\mu \tau }$ with the increments being the roots of the characteristic equation of system (A12) and (A13)

(A14) $$\mu_{1,2}=\delta -1+\frac{a^{2}}{2}\left(-1\pm \sqrt{1+\frac{2\delta^{2}}{a^{4}}}\right).$$

At large $a\;(a^{2}>>1)$, the increments are

(A15) $$\mu_{1}=\delta -1+\frac{\delta^{2}}{2a^{2}}.$$

(A16) $$\mu_{2}=\delta -1-a^{2}-\frac{\delta^{2}}{2a^{2}}.$$

The director field deformation threshold follows from (A15)

(A17) $$\delta =1-\frac{1}{8a^{2}}\approx 1.$$

At the threshold, as follows from (A12), (A13), and (A15), the ratio of mode amplitudes is

(A18) $$\frac{\psi_{1}}{\psi_{0}}=\frac{1}{a^{2}}.$$

To find the steady-state amplitudes, the nonlinear terms in Equations (A10) and (A11) should be taken into account. In this case ($\partial \psi_{0}/\partial \tau =0$,$\partial \psi_{1}/\partial \tau =0$), retaining in the system (A10), (A11) the terms $\sim \psi_{0}^{3}$ and $\psi_{1}\psi_{0}^{2}$, and subtracting (A11) from (A10), we find the estimate

(A19) $$\frac{\psi_{1}}{\psi_{0}}=\frac{1}{1+\frac{\delta }{2}+a^{2}-\frac{3}{4}\psi_{0}^{2}}\approx \frac{1}{a^{2}},$$

which coincides with (A18).

At a very high light intensity $\delta >>1+a^{2}$, the steady-state solution to the system (A10), (A11) is

(A20) $$\psi_{0}=\sqrt{2},\;\psi_{1}=0.$$

This solution corresponds to the uniform director field perpendicular to the electric field, the difference of $\sqrt{2}$ from π/2 is a consequence of the power expansion in Equation (A5). Experimentally, this situation starts to be implemented for the large beam power δ in the central part of the recorded pattern (see Figure 2g2).

Thus, the character of the director field in the oscillating field is defined by the parameter a according to estimate (A18).

The same conclusion can also be derived in the case of oblique incidence by using similar calculation procedure.

To compare the calculation results with the experiment, we should relate the mode amplitudes with the light-induced phase shift. To this end, we used an approximate formula for the phase S difference between the extraordinary and ordinary waves

(A21) $$S=k\delta n\int_{0}^{L}\sin^{2}\psi dy,$$

where δn is the optical anisotropy. Using (A7), (A9), and the approximation sinψ=ψ, we arrived at the equation

(A22) $$S\left(\psi_{0},\psi_{1},\xi \right)=\frac{S_{0}}{2}{\left(\psi_{0}-\psi_{1}\cos a\xi \right)}^{2},$$

where

(A23) $$S_{0}=k\delta nL,$$

is the phase shift between the extraordinary and ordinary waves when the director is rotated by an angle of 90°. A factor of 1/2 in Equation (A22) is due to the assumed approximation of the longitudinal dependence of director rotation ~sin(πy/L).

It follows from (A22) and (A23) that the difference between maximum and minimum phase shifts in the oscillating light field is

(A24) $$\Delta S=2S_{0}\psi_{0}\psi_{1},$$

while the spatially averaged phase shift is

(A25) $$\bar{S}=\frac{S_{0}}{2}\left(\psi_{0}^{2}+\frac{\psi_{1}^{2}}{2}\right).$$

We shall introduce the contrast of the phase pattern as

(A26) $$\kappa =\frac{\Delta S}{\bar{S}}.$$

From (A24) and (A25) it follows that

(A27) $$\kappa \approx 4\frac{\psi_{1}}{\psi_{0}}\approx \frac{4}{a^{2}}.$$

The estimate (A27) is very approximate and does not include the dependence on the light beam power and is incorrect at very high light power, when the director becomes uniform.
